# Inflammatory cytokine profiles in eyes with primary angle-closure glaucoma

**DOI:** 10.1042/BSR20181411

**Published:** 2018-12-07

**Authors:** Yayi Wang, Shida Chen, Yaoming Liu, Wenbin Huang, Xinyi Li, Xiulan Zhang

**Affiliations:** Zhongshan Ophthalmic Center, State Key Laboratory of Ophthalmology, Sun Yat-Sen University, Guangzhou 510060, China

**Keywords:** aqueous humor, cytokines, inflammation, primary angle-closure glaucoma

## Abstract

Acute primary angle-closure (APAC) eyes show an early ‘acute inflammatory’ condition, while the inflammation condition has not been fully elucidated in the development of primary angle-closure glaucoma (PACG). To evaluate the roles of inflammatory cytokines in the pathogenesis of PACG, this cross-sectional study involved 40 eyes of 32 PACG patients who required trabeculectomy and 24 eyes of 24 patients who required cataract surgery. The aqueous humor samples were collected at the time of surgery. Fifteen inflammatory cytokines were detected using the multiplex bead immunoassay technique, and the clinical information was recorded for the correlation analysis. Eight of the 15 cytokines were all detectable in both groups, including granulocyte colony-stimulating factor (G-CSF), interleukin (IL)-6, IL-8, monocyte chemotactic protein (MCP)-1, MCP-3, macrophage-derived chemokine (MDC), macrophage inflammatory protein (MIP)-1β, and vascular endothelial growth factor (VEGF). When compared with the cataract patients, the MCP-3, MDC, and VEGF levels were elevated in the PACG patients, while the MCP-1 and MIP-1β levels were decreased. However, the G-CSF, IL-6, and IL-8 levels were similar between the two groups. The MCP-1 concentration was elevated accordingly as the disease progressed in the PACG patients. Our results suggest the PACG eyes retained a ‘mild inflammation’ condition in the aqueous humor, and MCP-1 may play an important role in the progression of this disease.

## Introduction

Primary angle-closure glaucoma (PACG) is one of the main types of glaucoma leading to irreversible blindness, with a 1.1% prevalence rate in the Asian population [1]. It always exhibits an elevated intraocular pressure (IOP) because the aqueous humor outflow is blocked by the closed anterior chamber angle. When peripheral anterior synechiae (PAS) form between the peripheral iris and the trabecular meshwork, ocular hypertension gradually develops to an irreversible and uncontrolled extent, leading to glaucomatous optic nerve damage. Aqueous humor circulation homeostasis plays important roles in IOP maintenance and the physiological functions of the anterior segment eye structures.

Many studies have explored the changes in the cytokine and chemokine compositions in the aqueous humor in various ocular diseases, such as uveitis [2], branch retinal vein occlusion [3], age-related macular degeneration [4], and glaucoma [5–7]. In the aqueous humor of acute primary angle-closure (APAC) eyes, our previous findings showed an early ‘acute inflammatory’ condition, with significantly elevated concentrations of several inflammation-related cytokines, and the inflammation was relieved when the IOP decreased [5,8,9]. In eyes with chronic PACG, the IOP rising is gradual and lasts longer than in APAC. Therefore, the anterior chamber inflammatory responses are different between APAC and PACG patients. A few studies have revealed that the mean flare value and mean cell counts, as well as some of the aqueous cytokines, were slightly increased in the PACG patients when compared with the cataract controls [10–12], but other studies found no significant differences in the cytokines between the PACG group and the control group [5,13].

Until now, the inflammatory state in the PACG process has not been fully described, especially during the different disease stages. Therefore, the present study aimed to detect the inflammation-related cytokine levels in the aqueous humor of PACG eyes by using the multiplex bead immunoassay technique, which can detect many cytokines simultaneously with a small amount of aqueous fluid. In addition, the inflammatory conditions in the different disease stages, PAS extents and the influences of different anti-glaucoma drug choices were analyzed.

## Experimental

### Subjects

This cross-sectional study recruited 32 PACG patients who required trabeculectomy [14] and 24 patients requiring cataract surgery from the Zhongshan Ophthalmic Center at Sun Yat-sen University in Guangzhou, China. All the study participants received detailed explanations about the study, and they signed informed consent forms. This research was conducted in accordance with the principles embodied in the Declaration of Helsinki, and it was approved by the Ethical Review Committee at the Zhongshan Ophthalmic Center. All the subjects were from the Chinese Han population.

The diagnosis of PACG was defined as narrow angles (180° or more of iridotrabecular contact using indentation gonioscopy in the primary position), with glaucomatous optic neuropathy [defined as a vertical cup/disc (C/D) ratio > 0.7 and/or C/D asymmetry > 0.2 and/or focal notching of the neuroretinal rim], and compatible visual field defects measured using automated static perimetry (standard Swedish Interactive Testing Algorithm with a 24-2 test pattern) (Humphrey Visual Field Analyzer II; Carl Zeiss Meditec, Inc., Dublin, CA, U.S.A.) [15]. All the PACG patients enrolled in the present study required trabeculectomy due to the uncontrolled IOPs by anti-glaucoma drugs. The control group consisted of age-related cataract patients undergoing routine cataract surgeries without histories of other eye diseases or IOPs exceeding 21 mmHg. The PACG disease stage was defined according to the mean deviation (MD) values of the visual field: early stage with MD no worse than −6 decibels (dB), advanced stage with MD between −6 and −12 dB, and late stage with MD worse than −12 dB [16].

The exclusion criteria were any history of trauma, uveitis, or other intraocular or systemic inflammatory diseases; pre-existing ocular diseases, such as retinal artery/vein occlusion, diabetic retinopathy, and age-related macular degeneration; and any intraocular intervention, including paracentesis. Those eyes that required a trabeculectomy combined with cataract surgery or cataract surgery at a late date within the study period were also excluded.

### Aqueous humor collection

The aqueous humor samples (50–100 μl) were collected using the procedure described in our previous study [8]. The samples were collected during the paracentesis procedure at the beginning of the trabeculectomy or before the cataract surgery. All the samples were immediately frozen and stored at −80°C until the analyses were performed.

### Cytokine analysis

The cytokine concentrations were analyzed using a multiplex bead immunoassay system (Milliplex Human Cytokine kit; Millipore Corp., Billerica, MA, U.S.A.). The assays were performed according to the manufacturer’s instructions, and the samples were analyzed using a suspension array system (Bio-Plex 200; Bio-Rad Laboratories Inc., Hercules, CA, U.S.A.) [8]. The following 15 inflammatory cytokines were analyzed simultaneously: interleukin (IL)-1β, IL-6, IL-8, IL-10, granulocyte colony-stimulating factor (G-CSF), granulocyte macrophage colony-stimulating factor (GM-CSF), interferon (IFN)-γ, monocyte chemotactic protein (MCP)-1, MCP-3, macrophage-derived chemokine (MDC), macrophage inflammatory protein (MIP)-1α, MIP-1β, soluble CD40 ligand (sCD40L), tumor necrosis factor (TNF)-β, and vascular endothelial growth factor (VEGF). For each reaction, 25 μl of the aqueous humor sample was used. Based on the information provided by the manufacturer, the multiplex assay kit can quantitatively measure multiple cytokines from as little as 25 µl of bodily fluid. The detection limit for any analyte was 1 pg/ml, with a dynamic range of up to 10000 pg/ml.

### Statistical analysis

The data were processed and statistically analyzed using IBM SPSS Statistics for Windows, version 22.0 (IBM Corp., Armonk, NY, U.S.A.). For the categorical variables, the frequency distributions were calculated and compared using the χ^2^-test. For the numerical variables with a normal distribution, a two-sample independent *t*-test was performed, and the Mann–Whitney *U*-test was used when the numerical variables were not normally distributed. In order to test the differences in the concentrations of each cytokine among the different subgroups, a Kruskal–Wallis test was performed, followed by a least significant difference *t*-test to analyze the differences between two groups. *P* values of 0.05 were accepted as statistically significant. Correlations between cytokine concentrations and subjects’ clinical data, including age, IOP, and MD value, were calculated by Spearman’s correlation test. *P* < 0.0071 was accepted by Bonferroni correction for multiple comparisons.

## Results

The present study included 40 eyes of 32 PACG patients and 24 eyes of 24 cataract patients. The demographic and clinical characteristics are summarized in Table 1. The mean ages of the PACG patients and the controls were 57.3 ± 10.8 years old and 74.0 ± 5.5 years old, respectively (*P* < 0.001). There was no significant difference in the sex distribution between the two groups (*P* = 0.887). As expected, the PACG group had a higher mean IOP than the cataract group (*P* < 0.001).

**Table 1:** Demographic and clinical characteristics of the patients

Characteristics	PACG	Cataract	*P* value
Total no. of eyes	40	24	–
Age, mean (SD), year	57.3 (10.8)	74.0 (5.5)	<0.001^†^
Sex, no. of M/F	18/14	13/11	0.877^‡^
IOP^1^, mean (SD), mmHg	24.2 (10.5)	13.7 (3.5)	<0.001^^†^^
IOP^2^, mean (SD), mmHg	19.7 (7.6)	13.7 (3.5)^*^	<0.001^^†^^
MD, median (IQ)	−15.8 (23.4)	–	–
PSD, median (IQ)	5.5 (7.0)	–	–

Abbreviations: IOP^1^, the recorded highest intraocular pressure; IOP^2^, the intraocular pressure measured before surgery; IQ, interquartile range; MD, mean defect; PACG, primary angle-closed glaucoma; PSD, pattern standard deviation; SD, standard deviation.

* The IOP of cataract patients was only measured once before surgery, so the IOP^2^ is the same as IOP^1^.

† The differences of age and IOP between the two groups were tested by two-sample independent *t*-test.

‡ The difference of the sex distribution between the two groups was tested by χ^2^-test.

The concentrations of the 15 cytokines in the aqueous humor were measured in both groups (Table 2). Among them, only eight cytokines were all detectable in both groups, including G-CSF, IL-6, IL-8, MCP-1, MCP-3, MDC, MIP-1β, and VEGF. The MCP-3 (*P* = 0.012), MDC (*P* < 0.001), and VEGF (*P* = 0.033) concentrations were significantly higher in the PACG group than in the cataract group, while the MCP-1 (*P* = 0.021) and MIP-1β (*P* = 0.022) concentrations were significantly decreased in the aqueous humor of the PACG patients. However, the G-CSF, IL-6, and IL-8 concentrations did not differ significantly between the PACG group and the control group (G-CSF: *P* = 0.341; IL-6: *P* = 0.212; IL-8: *P* = 0.739).

**Table 2:** The levels of different cytokines in the aqueous

Cytokine	PACG	Cataract	*P* value^*^
G-CSF	1.0 (2.1)	1.4 (3.5)	0.341
GM-CSF	0	0	–
IFN-γ	0	0	–
IL-1β	0	0	–
IL-6	1.7 (3.6)	5.6 (28.9)	0.212
IL-8	5.8 (7.5)	6.7 (7.7)	0.739
IL-10	0	0	–
MCP-1	905.5 (569.0)	1154.5 (888.0)	**0.021**
MCP-3	3.2 (2.1)	0.5 (5.4)	**0.012**
MDC	33.5 (27.9)	7.1 (21.1)	**<0.001**
MIP-1α	0	0	–
MIP-1β	6.0 (9.8)	10.6 (10.7)	**0.022**
sCD40L	0	0	–
TNF-β	0	0	–
VEGF	104.0 (90.3)	71.1 (111.4)	**0.033**

Data are expressed as the median (interquartile range), pg/ml.

* The Mann–Whitney *U*-test was performed to compare the two groups.

The G-CSF and MCP-1 levels were significantly different among the different PACG disease stages (*P* = 0.017 and *P* = 0.026, respectively; Table 3). The G-CSF concentration was significantly higher in the advanced stage than in the early stage (*P* = 0.005), and it showed a downward trend in the later stage (*P* = 0.017). Moreover, the MCP-1 level was markedly increased in the late stage PACG eyes when compared with the early stage PACG eyes (*P* = 0.009). The other cytokines showed no significant changes when the disease progressed in the PACG patients. However, the different PAS extents had no effect on the cytokine levels (all *P* > 0.05, Table 4). Moreover, the prostaglandin use had an influence on the MIP-1β level in the PACG patients (*P* = 0.023, Table 5). Those patients taking prostaglandin had a higher MIP-1β level in the aqueous humor when compared with the patients undergoing other drug treatments (*P* = 0.007). However, the other cytokines did not differ significantly between the different medication groups (all *P* > 0.05). There was no significant correlation between the aqueous cytokine levels and age, the IOP measured before surgery or MD value (all *P* > 0.0071).

**Table 3:** The levels of cytokines in different disease stages

Cytokine	PACG-E (*n* = 17)	PACG-A (*n* = 11)	PACG-L (*n* = 12)	*P* value^*^
G-CSF	0.9 (1.3)	2.8 (21.7)	0.8 (2.4)	**0.017**
IL-6	1.5 (1.9)	3.6 (13.9)	1.7 (8.7)	0.159
IL-8	4.4 (3.8)	8.8 (13.5)	8.2 (5.7)	0.107
MCP-1	748.0 (365.0)	964.0 (685.0)	1068.5 (906.8)	**0.026**
MCP-3	3.2 (2.1)	3.2 (2.1)	3.2 (1.9)	0.957
MDC	36.4 (35.1)	25.7 (25.4)	34.5 (24.8)	0.725
MIP-1β	7.0 (8.3)	9.4 (9.5)	2.9 (5.8)	0.196
VEGF	99.3 (121.1)	104.0 (90.1)	126.5 (123.8)	0.459

Abbreviations: PACG-E, PACG in early stage; PACG-A, PACG in advanced stage; PACG-L, PACG in late stage.

Data are expressed as the median (interquartile range), pg/ml.

* Kruskal–Wallis test was performed to compare the three groups.

**Table 4:** The levels of cytokines in different extents of PAS

Cytokine	No synechia (*n* = 10)	≤1/2 synechia (*n* = 9)	>1/2 synechia (*n* = 21)	*P* value^*^
G-CSF	0.7 (2.7)	1.0 (1.4)	1.3 (3.7)	0.414
IL-6	2.0 (3.2)	1.5 (5.9)	1.8 (9.3)	0.926
IL-8	6.3 (12.4)	4.6 (3.7)	8.3 (7.3)	0.283
MCP-1	789.1 (520.5)	916.0 (392.5)	1008.0 (598.0)	0.350
MCP-3	3.7 (1.6)	3.2 (3.2)	3.2 (2.1)	0.547
MDC	44.8 (44.6)	26.3 (28.3)	35.4 (22.8)	0.564
MIP-1β	6.5 (15.2)	2.9 (8.4)	6.8 (9.7)	0.460
VEGF	94.3 (113.5)	84.7 (112.0)	121.0 (75.8)	0.270

Abbreviation: PAS, peripheral anterior synechiae.

Data are expressed as the median (interquartile range), pg/ml.

* Kruskal–Wallis test was performed to compare the three groups.

**Table 5:** The influence of anti-glaucoma drugs on the levels of cytokines

Cytokine	No-drugs (*n* = 11)	Combined (*n* = 15)	Prostaglandin (*n* = 14)	*P* value^*^
G-CSF	1.0 (1.3)	0.7 (1.4)	1.7 (9.1)	0.298
IL-6	1.6 (3.2)	1.5 (1.1)	4.2 (15.9)	0.106
IL-8	4.6 (3.2)	5.8 (5.3)	8.7 (7.1)	0.136
MCP-1	710.0 (644.0)	895.0 (361.0)	1042.5 (713.0)	0.290
MCP-3	3.2 (2.7)	3.2 (2.1)	4.2 (1.3)	0.461
MDC	27.5 (19.7)	43.1 (24.1)	30.5 (37.7)	0.458
MIP-1β	7.5 (10.1)	2.9 (4.9)	10.1 (11.8)	**0.023**
VEGF	99.3 (164.0)	94.8 (70.3)	116.5 (107.0)	0.575

Combined: other anti-glaucoma drugs exclude the prostaglandin.

Data are expressed as the median(interquartile range), pg/ml.

* Kruskal–Wallis test was performed to compare the three groups.

## Discussion

PACG carries a great risk of severe, permanent, bilateral visual impairment. Angle closure is a basic pathological process in PAGC, but the pathogenic mechanism has not been fully elucidated. We believe that the increased aqueous humor flow resistance and the aqueous humor quality changes influence the microenvironment of the anterior segment, ultimately leading to PACG deterioration. The present study used a multiplex bead immunoassay technique, a valid alternative method to ‘gold standard’, ELISA [17], to simultaneously detect multiple cytokines in a small volume of aqueous humor. The results revealed a ‘mild inflammation’ condition in the PACG eyes when compared with the cataract eyes. The MCP-3, MDC, and VEGF levels were elevated in the PACG patients when compared with the controls, but the MCP-1 and MIP-1β levels were decreased. Moreover, the MCP-1 concentration was elevated accordingly while the disease progressed in the PACG patients.

Previous studies have shown different aqueous humor inflammatory responses in angle closure eyes compared with various control eyes (Table 6). For example, Chua et al. found that the PACG group had higher levels of IL-8 and monokine induced by IFN-γ (CXCL9) than the cataract group [11]. Duvesh et al. reported that the IL-8, MIP-1β, eotaxin, and IFN-γ-induced protein (IP)-10 concentrations were higher, while the IL-9, IL-17, IL-5, TNF-α, and GM-CSF concentrations were lower in chronic PACG patients when compared with the cataract patient controls [12]. Although our study also found a difference in the inflammation-related cytokines between the PACG and cataract patients, the cytokine types differed from those of other studies. These differences may have been caused by the different disease stages of the patients enrolled in their studies, different ages, and different drug treatments, as well as the use of different multiplex bead systems from different vendors. However, all the studies including our studies showed there was a unique inflammatory response in the aqueous of PACG. In addition, anterior chamber inflammatory response was also found in several PACG animal models such as injecting hypertonic saline or latex microspheres, and episcleral vein cauterization model [18–20], suggesting inflammation was involved in PACG development.

**Table 6:** Aqueous cytokines of angle closure eyes in published literatures

Patient/control	Method	Different cytokine	Literature
APAC/PACS	Multiplex bead immunoassay	G-CSF, IL-6, IL-8, MCP-1, MCP-3, MDC, MIP-1β, VEGF↑	Du S, 2016
APAC/cataract	Multiplex bead immunoassay	IL-6, IL-8, G-CSF, MCP-1, MCP-3, VEGF↑	Huang W, 2014
APAC/cataract	Magnetic bead immunoassay, ELISA	sCD44 and VEGF↑	Chen S, 2015
APAC/cataract	Cytometric bead assay system	IL-2, IL-5, MCP-1, TNF-α, IP-10↑	Tong Y, 2017
APAC/cataract	Multiplex bead immunoassay	IL-12, IL-15, IL-6, IL-27↑	Liu YM, 2017
APAC/PACG	Multiplex bead immunoassay	MCP-1, MCP-3↑	Gao X, 2016
PACG/cataract	Multiplexed cytokine analysis	IL-8, CXCL9↑	Chua J, 2012
PACG/cataract	Multiplex bead immunoassay	IL-8, eotaxin, IP-10, MIP-1β↑ IL-9, IL-17, IL-5, TNF-α, GM-CSF↓	Duvesh R, 2017
PACG/cataract	Cytometric bead assay system	–	Tong Y, 2017
PACG/cataract	Multiplex bead immunoassay	MCP-3, MDC, VEGF↑ MCP-1, MIP-1β↓	Our study

Abbreviations: APAC, acute primary angle-closure; PACG, primary angle-closure glaucoma; PACS, primary angle-closure suspect.

Alternative activated (or M2) macrophages are the main sources of MDC. M2 macrophages play roles in tissue remodeling and inflammation reduction via their endocytic clearance capacity, trophic factor synthesis, and reduced pro-inflammatory cytokine secretion [21]. An MDC evaluation has been reported in several ocular diseases, such as wet age-related macular degeneration and unilateral APAC affected eyes [6,22]. However, to our knowledge, this is the first research study to perform an MDC evaluation using the aqueous humor of PACG eyes, suggesting M2 macrophage polarization and a self-tolerance function of the anterior tissue microenvironment. Moreover, VEGF plays important roles in angiogenesis and vascular permeability, which can greatly change the tissue microenvironment and induce inflammation [23]. Contrary to the sharp rise in the VEGF level of APAC eyes previously reported [6,8], the VEGF remained at a slightly higher level in the PACG eyes over a longer period time, indicating a hypoxic microenvironment in the anterior segment tissues of the PACG eyes. Finally, MCP-3 is one of the multifunctional chemokines with pro-inflammatory effects but often exhibits low expression, and it shares one of MCP-1 receptors with lower affinity than MCP-1 [24,25]. In our study, it was reasonable to examine both MCP-1 and MCP-3 as they are involved in activation and recruitment of monocytes to injury sites, and we found the MCP-3 level was just slightly raised in the PACG eyes.

MCP-1 and MIP-1β are typical inflammatory chemokines that participate in the inflammatory reaction by recruiting immune cells to the damaged area. In addition, MCP-1 plays a critical role in the healing pathway [26–28]; therefore, it may be a potential risk factor for the scar formation of a filtering bleb [5]. In our previous study, the APAC eyes showed significantly elevated MCP-1, MCP-3, MIP-1β, G-CSF, IL-6, and IL-8 concentrations when compared with the cataract group [5,8]. These aqueous mediators may leak due to the breakdown of the blood-aqueous barrier, or they may be locally produced by the inflamed anterior segment tissues. The evidence has suggested that an ‘acute inflammatory’ condition occurs in the APAC acute stage, and gradually, it decreases to a normal level in the previously APAC eyes. Interestingly, in the present study, we found decreased MCP-1 and MIP-1β concentrations and no differences in the G-CSF, IL-6, and IL-8 concentrations in the PACG eyes when compared with the cataract eyes, indicating a ‘mild inflammation’ condition in the aqueous humor. The different aqueous inflammatory conditions between the PACG and APAC eyes were consistent with the clinical manifestations, because PACG always develops quietly and insidiously. Previous studies have also compared the MCP-1 level of PACG eyes with that of cataract eyes, but no significant difference was observed [5,12,13], however, in our studies, lower MCP-1 concentration was detected in PACG than in cataract patients. The reason may be as follows: first, in previous studies, the cataract eyes with high myopia and diabetic retinopathy exhibited higher aqueous humor MCP-1 levels than the senile cataract eyes, and the MCP-1 level was elevated after phacoemulsification when compared with the preoperative eyes [29–31]. In our study, among the 24 cataract patients, about 13 patients had already had cataract surgery for the other eye before the aqueous humor were taken in the present study, which may cause a relative high concentration of MCP-1 detected in the cataract group. On the other hand, the normal trabecular meshwork endothelial cells constitutively secrete several factors, like MCP-1, which modulate the intracellular and extracellular environment to maintain the normal aqueous humor outflow pathway functions [21]. The routine secretion of these chemokines may be destroyed by the long-term blockage of the aqueous humor outflow in PACG.

After further separating the PACG group into different disease stages and PAS extents, we found that the MCP-1 level may have been related to the PACG progression. A positive correlation trend can also be observed between MCP-1 and MD value, but it failed to reach statistical difference probably because small sample size of the present study. MCP-1 may act as a biomarker to evaluate the PACG severity and a risk factor for filtering surgery failure in the late disease stages. Moreover, the G-CSF concentration increased in advanced stage PACG, but subsequently reduced in later stages. G-CSF could stimulate the survival, proliferation, differentiation, and function of granulocytes, and it has been correlated with an IOP elevation in APAC eyes [6]. However, the different PAS extents had no influence on the factor levels. The use of anti-glaucoma medications, such as prostaglandins which are pro-inflammatory molecules derived from arachidonic acid metabolism, may lead to conjunctival inflammation and influence the aqueous immune milieu [32]. In the present study, the PACG patients using prostaglandin exhibited a higher MIP-1β level in the aqueous humor than in the other patients under drug treatment. These results support a previous flare cell study that treatment with prostaglandin analogs may induce a subclinical anterior chamber inflammation [33]. Overall, the disease stages and the treatments are both important factors that influence the inflammatory condition in PACG eyes (Table 7 showing the treatments of different PACG stages patients), but they might influence different cytokines and signaling pathways.

**Table 7:** Treatments of different PACG stages patients

Stage	Treatment		
	No-drugs	Combined	Prostaglandin
PACG-E	9	6	2
PACG-A	1	2	8
PACG-L	1	7	4

Number represents the number of eyes.

The present study did have some limitations. First, only 40 PACG eyes were enrolled, but the results were statistically significant, which probably strengthens the conclusions. Secondly, healthy people are actually the best control group, however, it is improbable to collect their aqueous humor and this invasive procedure may not be approved by the Ethical Review Committee. So, we used cataract patients who required routine surgery as noninflammatory controls based on previous research experiences. And in the present study, the cytokine levels in the cataract aqueous were relatively low, which were similar with previous studies. Thirdly, the mean age of the cataract group was older than that of the PACG group. We enrolled cataract patients with larger age because they were less likely to develop PACG in later life. Besides, no significant correlation was found between the aqueous cytokine levels and age, as along with most previous studies [5–8,11,12]. Last, we could not confirm the exact reasons for the changing inflammatory cytokines because we only measured the concentrations. Further studies using anterior segment tissue samples from PACG patients or glaucoma animal models will help to explore the exact roles of inflammation in the pathogenesis of PACG.

In summary, the present study showed that some cytokines related to inflammation were changed in PACG eyes when compared with cataract eyes, including elevated MCP-3, MDC, and VEGF levels and reduced MCP-1 and MIP-1β levels. Moreover, MCP-1 may play an important role in the progression of this disease. The exact reasons for the ‘mild inflammation’ aqueous condition and the specific functions of each cytokine in the pathogenesis and progression of PACG require further investigation.

## Clinical perspectives

Currently, the inflammatory state in the PACG has not been fully explored.

In the present study, we found the levels of MCP-3, MDC, and VEGF were elevated while the levels of MCP-1 and MIP-1β were decreased in PACG eyes than cataract eyes. Moreover, the MCP-1 level was increased in the late stage compared with the early stage PACG eyes.

These results suggest that the PACG eyes retained a chronic ‘mild inflammation’ condition in the aqueous humor, and MCP-1 may play an important role in the progression of this disease. Anti-inflammation may serve as a potential therapy to PACG.

## Abbreviations

APAC: acute primary angle-closure
C/D ratio: cup/disc ratio
CXCL9: monokine induced by IFN-γ
dB: decibels
G-CSF: granulocyte colony-stimulating factor
GM-CSF: granulocyte macrophage colony-stimulating factor
IFN-γ: interferon-γ
IL-1β: interleukin-1β
IL-6: interleukin-6
IL-8: interleukin-8
IL-10: interleukin-10
IOP: intraocular pressure
IP-10: IFN-γ-induced protein-10
M2 macrophages: alternative activated macrophages
MCP-1: monocyte chemotactic protein-1
MCP-3: monocyte chemotactic protein-3
MD: mean deviation
MDC: macrophage-derived chemokine
MIP-1α: macrophage inflammatory protein-1α
MIP-1β: macrophage inflammatory protein-1β
PACG: primary angle-closure glaucoma
PAS: peripheral anterior synechiae
sCD40L: soluble CD40 ligand
TNF-β: tumor necrosis factor-β
VEGF: vascular endothelial growth factor
